# Comparative analysis of the medicinal plant *Polygonatum kingianum* (Asparagaceae) with related verticillate leaf types of the *Polygonatum* species based on chloroplast genomes

**DOI:** 10.3389/fpls.2023.1202634

**Published:** 2023-08-23

**Authors:** Naixing Shi, Zefen Yang, Ke Miao, Lilei Tang, Nian Zhou, Pingxuan Xie, Guosong Wen

**Affiliations:** ^1^ College of Agronomy and Biotechnology, Yunnan Agricultural University, Kunming, China; ^2^ Chinese Academy of Sciences (CAS) Key Laboratory for Plant Diversity and Biogeography of East Asia, Kunming Institute of Botany, Chinese Academy of Sciences, Kunming, China; ^3^ College of Traditional Chinese Medicine, Guangdong Pharmaceutical University, Guangzhou, China

**Keywords:** *Polygonatum*, verticillate leaf, *Polygonatum kingianum*, chloroplast genome, comparative analysis, phylogenetic analysis

## Abstract

**Background:**

*Polygonatum kingianum* has been widely used as a traditional Chinese medicine as well as a healthy food. Because of its highly variable morphology, this medicinal plant is often difficult to distinguish from other related verticillate leaf types of the *Polygonatum* species. The contaminants in *P. kingianum* products not only decrease the products’ quality but also threaten consumer safety, seriously inhibiting the industrial application of *P. kingianum*.

**Methods:**

Nine complete chloroplast (cp) genomes of six verticillate leaf types of the *Polygonatum* species were *de novo* assembled and systematically analyzed.

**Results:**

The total lengths of newly sequenced cp genomes ranged from 155,437 to 155,977 bp, including 86/87 protein-coding, 38 tRNA, and 8 rRNA genes, which all exhibited well-conserved genomic structures and gene orders. The differences in the IR/SC (inverted repeats/single-copy) boundary regions and simple sequence repeats were detected among the verticillate leaf types of the *Polygonatum* cp genomes. Comparative cp genomes analyses revealed that a higher similarity was conserved in the IR regions than in the SC regions. In addition, 11 divergent hotspot regions were selected, providing potential molecular markers for the identification of the *Polygonatum* species with verticillate leaf types. Phylogenetic analysis indicated that, as a super barcode, plastids realized a fast and efficient identification that clearly characterized the relationships within the verticillate leaf types of the *Polygonatum* species. In brief, our results not only enrich the data on the cp genomes of the genus *Polygonatum* but also provide references for the *P. kingianum* germplasm resource protection, herbal cultivation, and drug production.

**Conclusion:**

This study not only accurately identifies *P. kingianum* species, but also provides valuable information for the development of molecular markers and phylogenetic analyses of the *Polygonatum* species with verticillate leaf types.

## Introduction

*Polygonatum kingianum* belongs to the perennial herb of *Polygonatum* Mill in Asparagaceae. Its wild resource distributes widely in southwest China and southeast Asia (Myanmar, Vietnam) (Rix and Rushforth, 2016; Duan et al., 2017). Because of its great medicinal and edible value, *P. kingianum*, a top medicinal herb, is listed in the Chinese ancient medical book (Ming Yi Bie Lu, Han Dynasty, 220-450 AD) with the Chinese name “Huangjing”. Modern pharmacological studies have shown that its components possess anti-aging, anti-tumor, immune enhancement, sterilization, and anti-inflammatory effects (Zhao and Li, 2015; Zhao et al., 2018b; Zhang et al., 2019). Based on its important value, hundreds of commercial drugs and health products whose raw materials include *Polygonati rhizome* have been developed in China (Su et al., 2018).

The genus *Polygonatum* is roughly divided into verticillate leaf types of the *Polygonatum* species (*P.* sect. *Sibirica* + *P.* sect. *Verticillata*) and alternate phyllotaxis *Polygonatum* species (*P.* sect. *Polygonatum*) (Liu, 2018; Peng, 2018). According to the description of the genus *Polygonum* in the Flora of China (FOC), the verticillate leaf type of the *Polygonatum* species holds a more variable morphology than the alternate phyllotaxis *Polygonatum* species, and even some morphological intermediates of the species have been found. *P. kingianum* is a typical representative of the verticillate leaf type of the *Polygonatum* species. However, the highly variable morphology of *P. kingianum* makes it difficult to distinguish it from the other verticillate leaf types of the *Polygonatum* species. Previous surveys have revealed that *P. kingianum* is generally contaminated with common adulterants, such as *P. zanlanscianense, P. cirrhifolium, P. verticillatum*, and other verticillate leaf types of the *Polygonatum* species (Liu, 2018; Zhang et al., 2023). These adulterants are usually of poor quality and some may even impair the clinical safety and efficacy of *P. kingianum* (Wang et al., 2019). Although the phylogenetic relationships of the *Polygonatum* genus have been reported recently, some research results were inconsistent, especially regarding the verticillate leaf type of the *Polygonatum* species (Zhao et al., 2018a; Wang et al., 2022; Xia et al., 2022).

Chloroplast (cp) is an important subcellular organelle for photosynthesis and energy transformation in plant cells (Verma and Daniell, 2008). In addition, cp holds a relatively independent genome. In most land plants, the cp genome belongs to the maternal inheritance, which has the characteristics of stable structure, conserved coding region sequence, and rich information (Wolfe et al., 1987; Wicke et al., 2011). Compared with standard DNA barcodes and ultra-barcodes, plastids can provide more abundant genetic variation information and higher species discrimination capabilities (Hollingsworth et al., 2016). With high throughput sequencing methods, the cp genome has been widely used in plant system evolution, related species identification, genetic diversity analysis, cp genetic engineering, etc. (Zhao et al., 2018c; Li L. et al., 2021; Fang et al., 2023). Therefore, it is of great significance to accurately identify *P. kingianum* and clarify the complex genetic background of the verticillate leaf type of the *Polygonatum* species. The use of the cp genome can improve the safety and effectiveness of clinical drug use and the development and utilization of *Polygonatum* medicinal plants.

In the present study, we reported on the whole cp genomes of six verticillate leaf types of the *Polygonatum* species. These sequences together with earlier published *Polygonatum* cp genome data helped us better understand its genome structure, codon usage preference, repeat sequences, mutation hotspots, and phylogenetic relationships. The data acquired in this study not only increased the genomic resources available for the *Polygonatum* genus but also provided valuable information for the phylogenetic analysis and identification of the verticillate leaf type of the *Polygonatum* species, as well as for the safe medical applications of *P. kingianum*.

## Materials and methods

### Plant samples and DNA extraction

*P. kingianum* with different morphological characteristics and other verticillate leaf-type *Polygonatum* species were obtained from Yunnan Province, China (**Supplementary Table 1**). Fresh and healthy leaves from these plants were collected and stored in silica gel for DNA extraction. The voucher specimens were identified by Professor Yunheng Ji and deposited in the herbarium of Kunming Institute of Botany (Chinese Academy of Sciences). In addition, 12 *Polygonatum* plastomes were downloaded from the NCBI database for subsequent analysis (**Supplementary Table 2**).

### Genome sequencing, assembly, and annotation

Total DNA was extracted from approximately 20 mg silica-gel dried leaf tissues using the modified CTAB method (Porebski et al., 1997). Sequence libraries were constructed using approximately 5 µg of purified genomic DNA with a TruSeq DNA Sample Prep Kit (Illumina, Inc., San Diego, CA, United States) according to the manufacturer’s recommendation. The NGS QC tool kit tool was used to trim the raw reads to obtain clean data (Patel and Jain, 2012).

We used NOVOPlasty v4.1.0 to perform cp genome assembly with a k-mer of 39 (Nicolas et al., 2017). Gene *matK* sequence from *P*. *kingianum* (GenBank: JX185477) as the seed for iterative extension of contigs to recover the whole plastome of each accession. The positions of start and stop codons were adjusted manually with a BLAST search against the NCBI protein database. All tRNA genes were identified by the tRNAscan-SE 2.0 with default parameters (Schattner et al., 2005). Thereafter, the boundaries of IR (inverted-repeat) and SC (single-copy) regions were defined by Geneious v. 9.0.2 (Kearse et al., 2012). Organellar Genome DRAW (Lohse et al., 2007) was used to draw the cp genome maps (http://ogdraw.mpimp-golm.mpg.de/). Additionally, all annotated cp genome sequences were submitted to GenBank on the NCBI website (https://www.ncbi.nlm.nih.gov/).

### Comparative chloroplast genome analysis

To verify the expansion or contraction of cp gene regions within verticillate leaf type of the *Polygonatum* species, the alignment was visualized using online mVISTA (https://genome.lbl.gov/vista/index.shtml) (Mayor et al., 2000) in Shuffle LAGAN mode, with the annotated cp genome of *P. kingianum* (Genbank number: MW566455) as a reference.

The protein-coding genes and intergenic spacers of the 20 plastomes (16 *Polygonatum* species) were extracted in Geneious v9.0.2 (Kearse et al., 2012) and aligned using the MAFFT v7.221 software (Katoh and Standley, 2013). Then, nucleotide diversity values (Pi) were calculated to estimate the divergence level of shared genes and intergenic spacers using DnaSP v5 (Librado and Rozas, 2009). The IRscope online program (https://irscope.shinyapps.io/irapp/) was used to evaluate IR expansion and contraction of plastome genetic architecture (Amiryousefi et al., 2018).

### SSRs and codon usage bias analysis

Online program (https://webblast.ipk-gatersleben.de/misa/): MISA software was used to detect simple sequence repeats (SSRs) in the cp genomes (Beier et al., 2017). Parameter setting thresholds of 10, 5, 4, 3, 3, and 3 were set for mono-, di-, tri-, tetra-, penta-, and hexanucleotide SSRs, respectively. 

The amount of codon and relative synonymous codon usage (RSCU) were calculated by CodonW v.1.4.2 with default parameters. Moreover, the heatmap of the RSCU values was shown by using TBtools software (Chen et al., 2020). Following the previous research methods, protein-coding genes (PCGs) of less than 300 nucleotides in length and the repetitive gene sequences were removed to reduce the deviation of the results (Fang et al., 2023).

### Phylogenetic analysis

All 21 available plastome sequences, including 12 published sequences downloaded from NCBI and 9 sequences obtained in this study, were included in the phylogenetic analysis. We constructed the phylogenetic trees by Maximum likelihood (ML) and Bayesian inference (BI) using the entire cp genomes. *P. cyrtonema*, the relative of *P. kingianum*, was used as the outgroup to root the phylogenetic trees. The ML tree was built using the CIPRES Science Gateway website (https://www.phylo.org/) under a General Time Reversible + Gamma (GTR + G) model and estimated with 1,000 bootstrap replicates (Stamatakis, 2006). The BI analysis was performed with Mrbayes 3.2.7 under the best substitution models and parameters (Posada and Crandall, 1998; Ronquist and Huelsenbeck, 2003; Posada and Buckley, 2004). The resulting phylogenetic trees were visualized by FigTree v1.4.4 software (http://tree.bio.ed.ac.uk/software/figtree/).

## Results

### Features of nine sequenced chloroplast genomes

**Figure 1:**
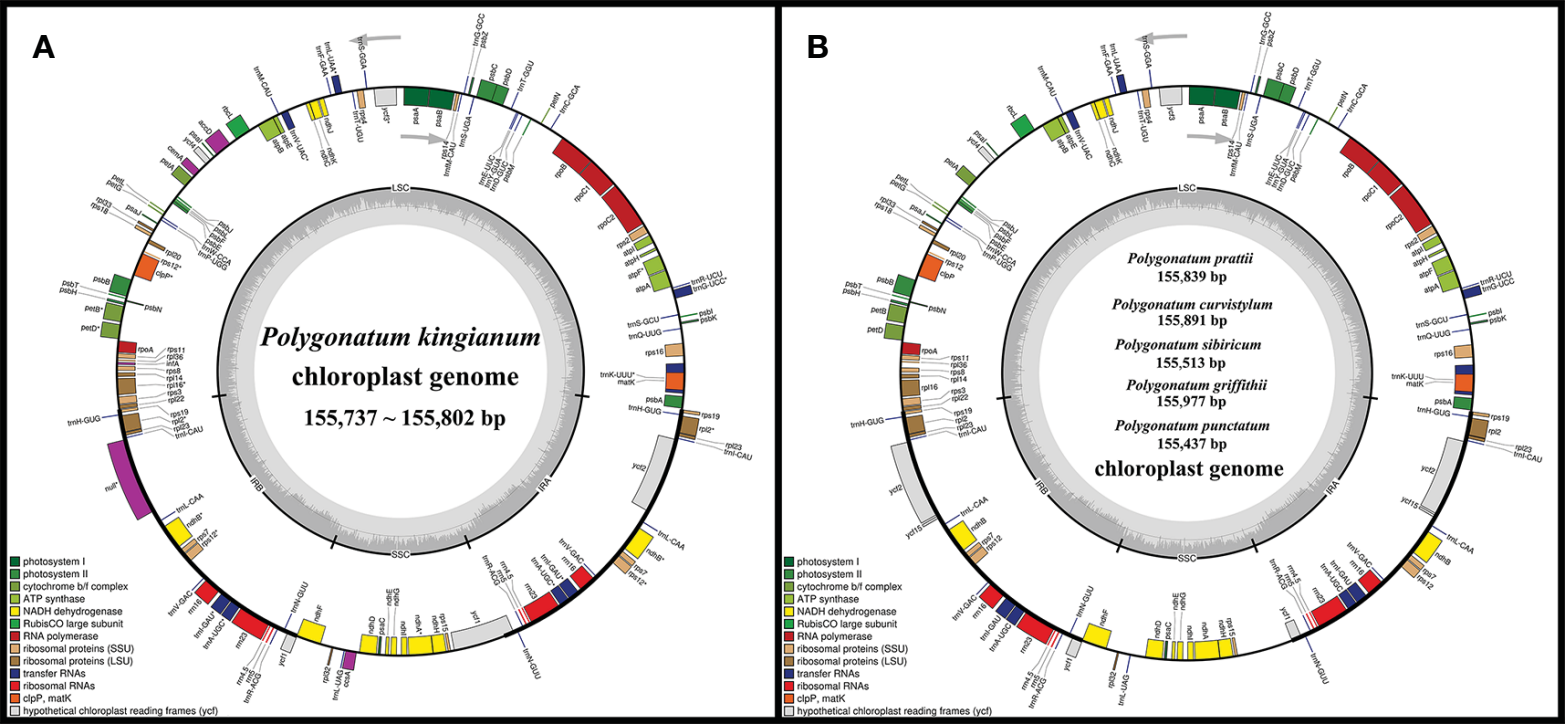
**(A)** Physical map of the four P. kingianum chloroplast genomes. **(B)** Chloroplast genome maps of *P. prattii, P. curvistylum, P. sibiricum, P. griffithii* and *P. punctatum*. Genes shown outside the circle are transcribed clockwise, while those inside are transcribed counterclockwise. Genes belonging to different functional groups are distinguished by color coding.

The size of newly sequenced plastid genomes within this study varied from 155,437 to 155,977 bp (**Supplementary Table 1**). All of them exhibited typical quadripartite structures that were similar to the pattern observed in other typical cp genomes of angiosperms, consisting of a pair of inverted repeat regions from 26,278 bp to 26,394 bp, a long single copy region 84,406 bp to 84,742 bp, and a short single copy region 18,415 bp to 18,554 bp (**Supplementary Table 1**; **Figure 1**). The GC contents of the nine plastomes were very similar and ranged from 37.6% to 37.7% (**Supplementary Table 1**). In this study, 133 genes were annotated in these cp genomes, including 86–87 protein-coding genes, 38 genes encoding transfer RNA, and 8 genes encoding ribosomal RNA. All encoded genes were divided into four categories according to their functions, including self-replication genes, genes related to photosynthesis, other genes, and unknown function genes (**Table 1**). Among these genes, 18 genes were intron-containing genes, 15 genes containing one intron (*rpl2*, *rpl16*, *rpoC1*, *rps16*, *trnA*-*UGC*, *trnG*-*UCC*, *trnI-GAU*, *trnK*-*UUU*, *trnL-UAA*, *trnV-UAC*, *atpF*, *ndhA*, *ndhB*, *petB*, and *petD*), and others containing two introns (*rps12*, *clpP*, and *ycf3*) (**Table 1**). All *infA* genes were found as pseudogenes in verticillate leaf types of the *Polygonatum* species except the *P. sibiricum*. This is because a G base was inserted into the *infA* sequence, which caused the *infA* gene to contain in-frame stop codons. All sample cp genome sequences were deposited in the GenBank database of the NCBI (**Supplementary Table 1**).

**Table 1:** List of annotated genes in the newly sequenced chloroplast genomes of the *Polygonatum* species.

Category of Genes	Group of gene	Name of gene
Self-replication	Ribosomal RNA genes	*rrn4.5*×2, *rrn5*×2, *rrn16*×2*, rrn*23×2
	Transfer RNA genes	trnC-GCA, trnD-GUC, trnE-UUC, trnF-GAA, trnG-UCC*, trnG-GCC, trnH-GUG×2, trnK-UUU*, trnL-UAA*, trnL-UAG, trnM-CAU, trnP-UGG, trnQ-UUG, trnR-UCU, trnS-GCU, trnS-GGA, trnS-UGA, trnT-UGU, trnT-GGU, trnV-UAC*, trnY-GUA, trnW-CCA, trnfM-CAU, trnA-UGC* ×2*, trnI-CAU×*2, trnI-GAU*×2*, trnL-CAA*×2*, trnN-GUU*×2, *trnR-ACG*×2*, trnV-GAC*×2
	Ribosomal protein (small subunit)	*rps2, rps3, rps4, rps7*×2, rps8, rps11, rps12**×2, rps14, rps15, rps16*, rps18, rps19×2
	Ribosomal protein (large subunit)	rpl2*×2, rpl14, rpl16*, rpl20, rpl22, rpl23×2*, rpl32, rpl33, rpl36*
	RNA polymerase	rpoA, rpoB, rpoC1*, rpoC2
Genes for photosynthesis	Subunits of photosystem I	psaA, psaB, psaC, psaI, psaJ, ycf3**, ycf4
	Subunits of photosystem II	*psbA, psbB, psbC, psbD, psbE, psbF, psbH, psbI, psbJ, psbK, psbL, psbM, psbN, psbT, psbZ*
	Subunits of cytochrome	petA, petB*, petD*, petG, petL, petN
	Subunits of ATP synthase	atpA, atpB, atpE, atpF*, atpH, atpI
	Large subunit of Rubisco	*rbcL*
	Subunits of NADH dehydrogenase	ndhA*, ndhB*×2*, ndhC, ndhD, ndhE, ndhF, ndhG, ndhH, ndhI, ndhJ, ndhK*
Other genes	Translational initiation factor	*infA*
	Maturase	*matK*
	Envelope membrane protein	*cemA*
	Subunit of acetyl-CoA	*accD*
	Synthesis gene	*ccsA*
	ATP-dependent protease	clpP**
	Component of TIC complex	*ycf1*×2
Genes of unknown function	Conserved open reading frames	*ycf2*×2, *ycf3*, *ycf4*

×2, Two gene copies in IR regions; *, With one intron; **, With two introns.

### IR expansion and contraction

**Figure 2:**
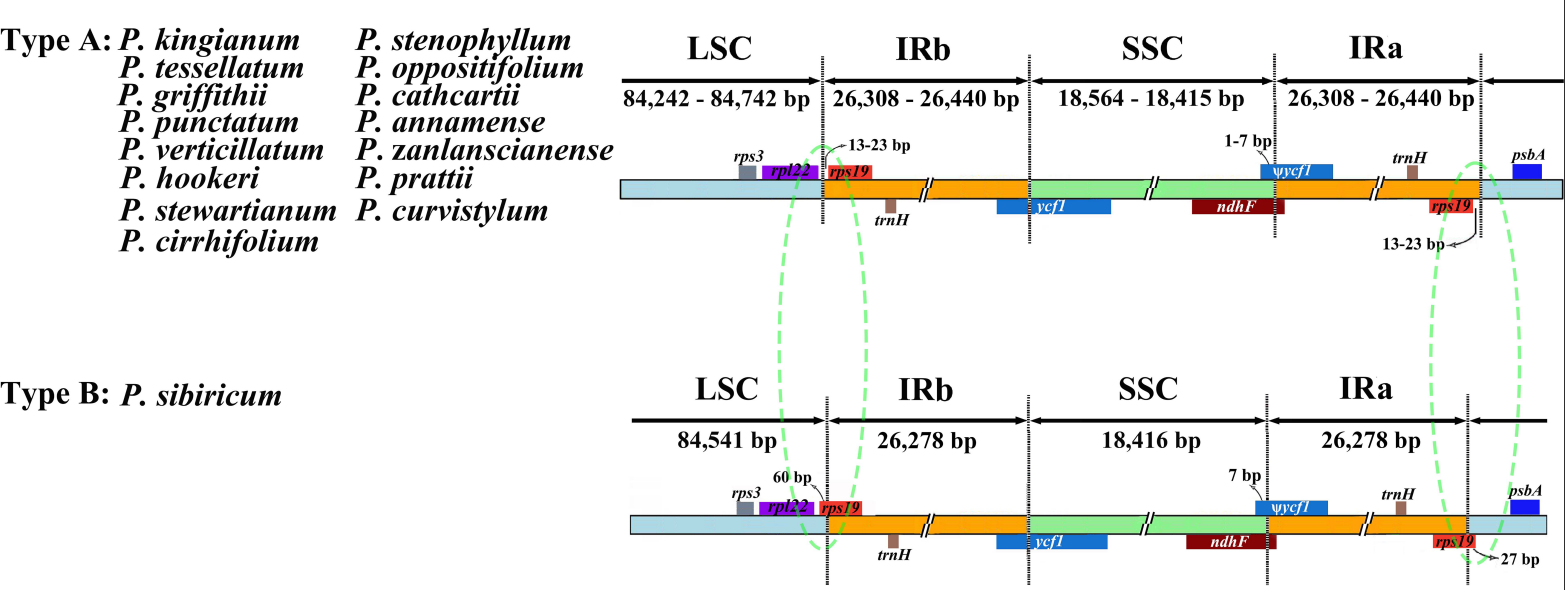
Comparison of LSC, SSC, and IR region boundaries in 16 verticillate leaf types of the *Polygonatum* species chloroplast genomes. LSC, large single-copy; SSC, small single-copy; IR, inverted repeat. Colored boxes denote genes. The distance between the genes and the boundaries is indicated by the base lengths (bp).

The expansions and contractions of IR borders were considered to be evolutionary events and the main cause of cp genome length changes (Wang et al., 2008). We analyzed the border regions of 16 verticillate leaf types of *Polygonatum* species plastomes to find similarities and differences (**Figure 2**). In our study, the *rps19* and *rpl22* genes were located in the IRb/LSC borders. In the SSC/IRa border, *rps19* and *psbA* genes were found. The *ndhF* crossed the IRa/SSC region. The *ycf1* gene was a cp-genome-encoded giant open reading frame that crossed the IRb/LSC region. Both the pseudogene fragment ψ*ycf1* and the *ndhF* gene were located at the IRb/SSC border and partially overlapped in the verticillate leaf type of the *Polygonatum* species. In *P. sibiricum*, the two *rps19* genes extended into the LSC region by 27 and 60 bp, respectively. However, the *rps19* genes of other species were entirely located in the IR regions.

### Plastome comparison

**Figure 3:**
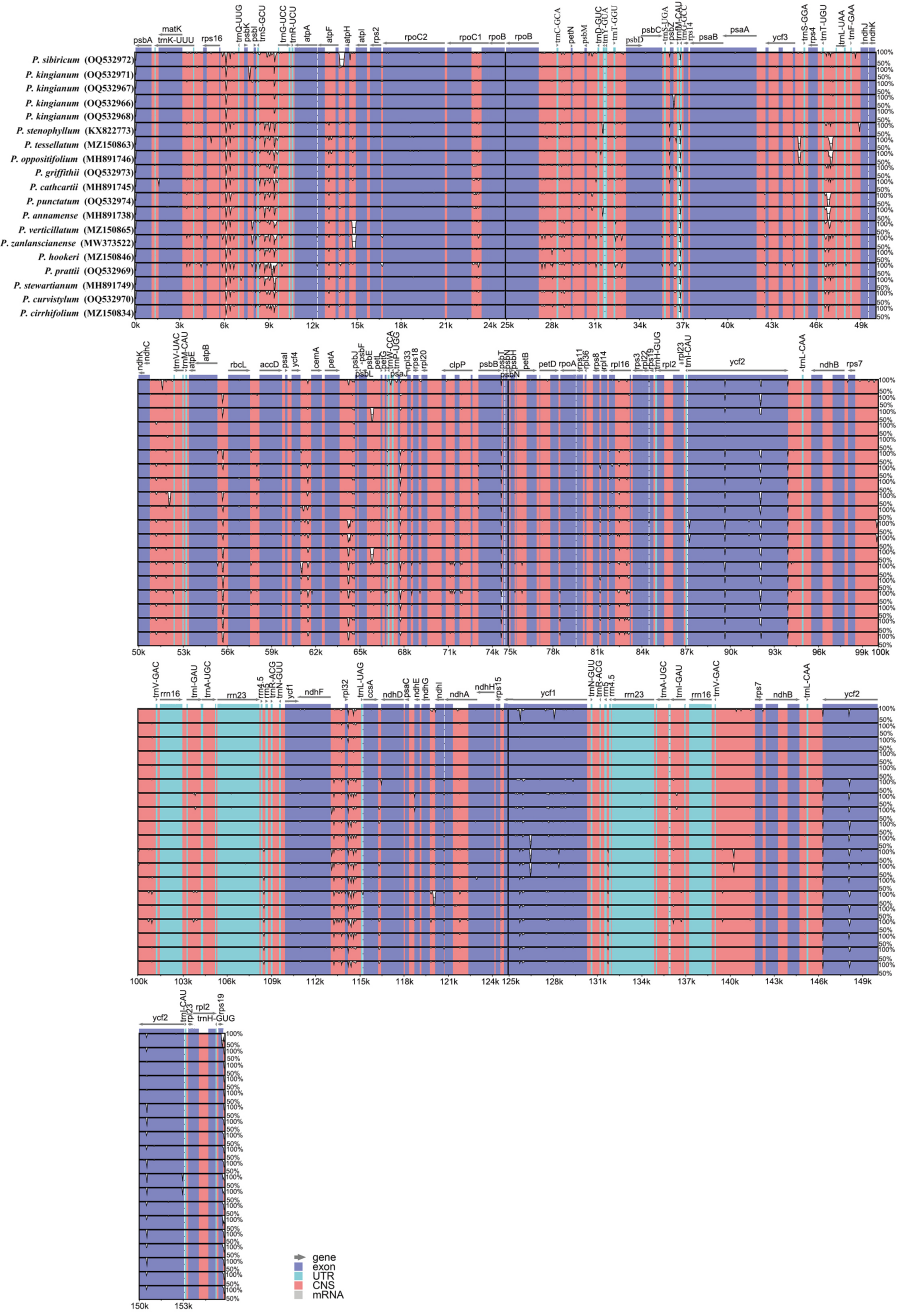
Alignment of the verticillate leaf type of the *Polygonatum* species chloroplast genome sequences with mVISTA using *P. kingianum* (Genbank number: MW566455) annotation as a reference. Exon, untranslated region (UTR), conserved noncoding sequences (CNS), and mRNA are color-marked. The direction of the gene transcription is indicated by gray arrows. The vertical scale represents the percentage of identity, ranging from 50 to 100%.

In order to further analyze the results, the 16 verticillate leaf types of the *Polygonatum* species (20 plastid sequences) were aligned and visualized to determine the overall variations by the program mVISTA. In agreement with other studies (Huang et al., 2021; Wu et al., 2021; Yang et al., 2021), the result of the alignment revealed that the coding regions are more highly conserved than non-coding regions. Most of the variations detected were found in non-coding sequence areas (**Figure 3**).

**Figure 4:**
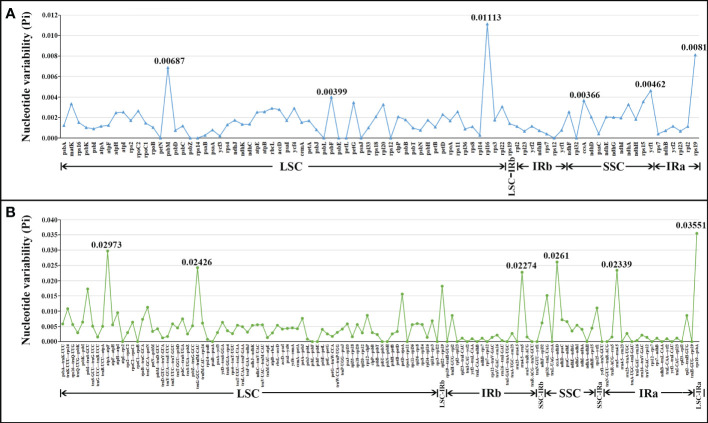
Comparative analysis of the nucleotide diversity (Pi) values among the 20 plastomes (16 *Polygonatum* species): **(A)** protein-coding genes; **(B)** non-coding and intron regions.

The nucleotide polymorphism (Pi) values of the 20 cp genomes in this study were computed to detect hyper-variable regions using the DnaSP v5 software (Librado and Rozas, 2009). **Figure 4** shows that the IR regions were more conserved than the LSC and SSC regions. The average Pi value of shared PCGs ranged from 0 to 0.01113, and the average Pi value of intergenic spacers ranged from 0 to 0.03551 (**Supplementary Table 3**; **Figure 4**). These hotspots (PCGs, Pi > 0.0036; Intergenic spacers, Pi > 0.02) were selected including *ccsA*, *psbF*, *ycf1*, *psbM*, *rps19*, *rpl16*, *rrn4.5–rrn5*, *trnG-GCC–trnfM-CAU*, *ccsA–ndhD*, *atpA–atpF*, and *rps19–psbA*, which were potentially useful for species identification in *P. kingianum* with related verticillate leaf type of the *Polygonatum* species.

### SSRs and codon usage analysis

**Figure 5:**
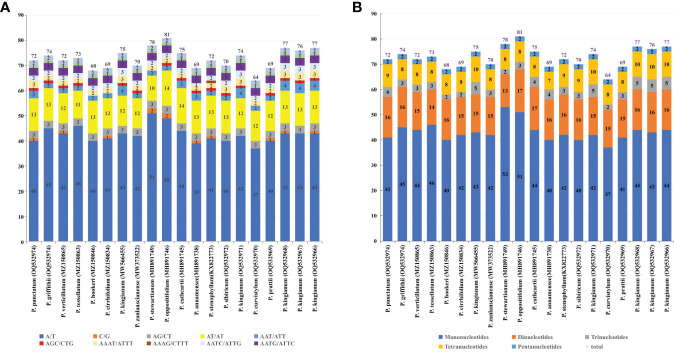
Simple sequence repeats (SSRs) in the 20 cp genomes. **(A)** Distribution of SSR repeat units in the 20 cp genomes; **(B)** Distribution of six different types of SSRs in the 20 cp genomes.

SSRs are useful for detecting genetic diversity and polymorphisms at the population, intraspecific, and cultivar levels, as well as for species-distinguishing (Zaloglu et al., 2015; Guo et al., 2018; Guang et al., 2019). The total number of simple sequence repeats were identified in cp genomes by Perlscript MicroSAtellite (MISA) (Beier et al., 2017) and ranged from 64 to 81, with the most abundant type of SSRs being the mono-nucleotide repeats (**Figure 5B**; **Supplementary Table 4**). In each case, the 37–53 mononucleotide SSRs, 13–17 dinucleotide SSRs, 2–5 trinucleotide SSRs, 7–10 tetranucleotide SSRs, and 2 pentanucleotides SSRs were identified. There were no hexanucleotide repeats detected in the verticillate leaf type of the *Polygonatum* species. Most repeats of all six microsatellite types were of the A/T motif rather than the G/C motif (**Figure 5A**; **Supplementary Table 4**). SSRs composed of G/C were not found in the plastomes of *P. griffithii*, *P. tessellatum*, *P. hookeri*, *P. zanlanscianense*, *P. cathcartii*, *P. sibiricum*, *P. curvistylum*, *P. kingianum* (OQ532971), *P. kingianum* (MW566455), and *P. kingianum* (OQ532967), while trinucleotide SSR AGC/CTG was only present in *P. curvistylum*, *P. stewartianum*, *P. hookeri*, and *P. cirrhifolium.* The tetranucleotide SSR AAAG/CTTT was only detected in the cp genome of the *P. stenophyllum*.

**Figure 6:**
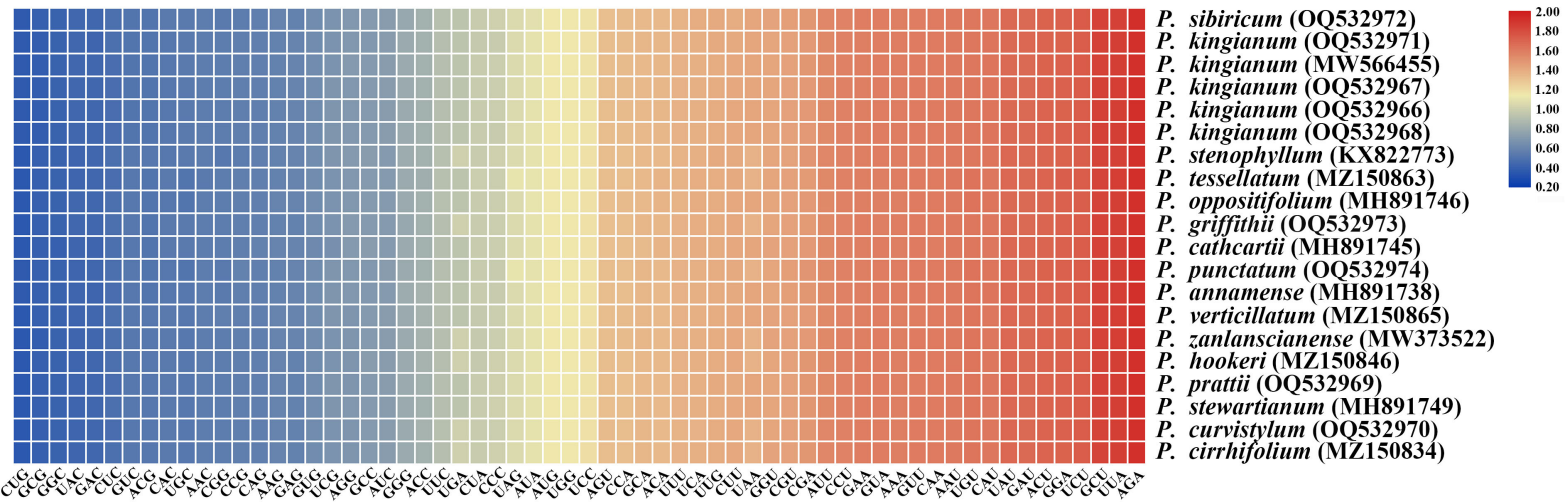
Heat map of the RSCU values of the 20 plastomes in the 16 verticillate leaf types of the *Polygonatum* species. Color key: Red and blue indicate higher and lower RSCU values, respectively.

A total of 56 protein-coding genes were selected for codon usage analysis. A total of 64 codons were observed, of which 61 codons encoded 20 amino acids and 3 stop codons (**Figure 6**; **Supplementary Table 5**). These sequences were 72,513–72,885 bp in length and encoded 24,171–24,295 codons. Of these amino acids, leucine had the highest number of codons, whereas cysteine had the least (**Supplementary Table 5**). **Figure 6** presents the relative synonymous codon usage (RSCU) of each plastome. RSCU values differ slightly in these plastid sequences. Additionally, the results showed that the RSCU values of 31 codons were higher than 1 and that 29 codons presented A/U- endings. Tryptophan and methionine were the amino acids without codon bias (RSCU = 1).

### Species identification and phylogenetic analysis

**Figure 7:**
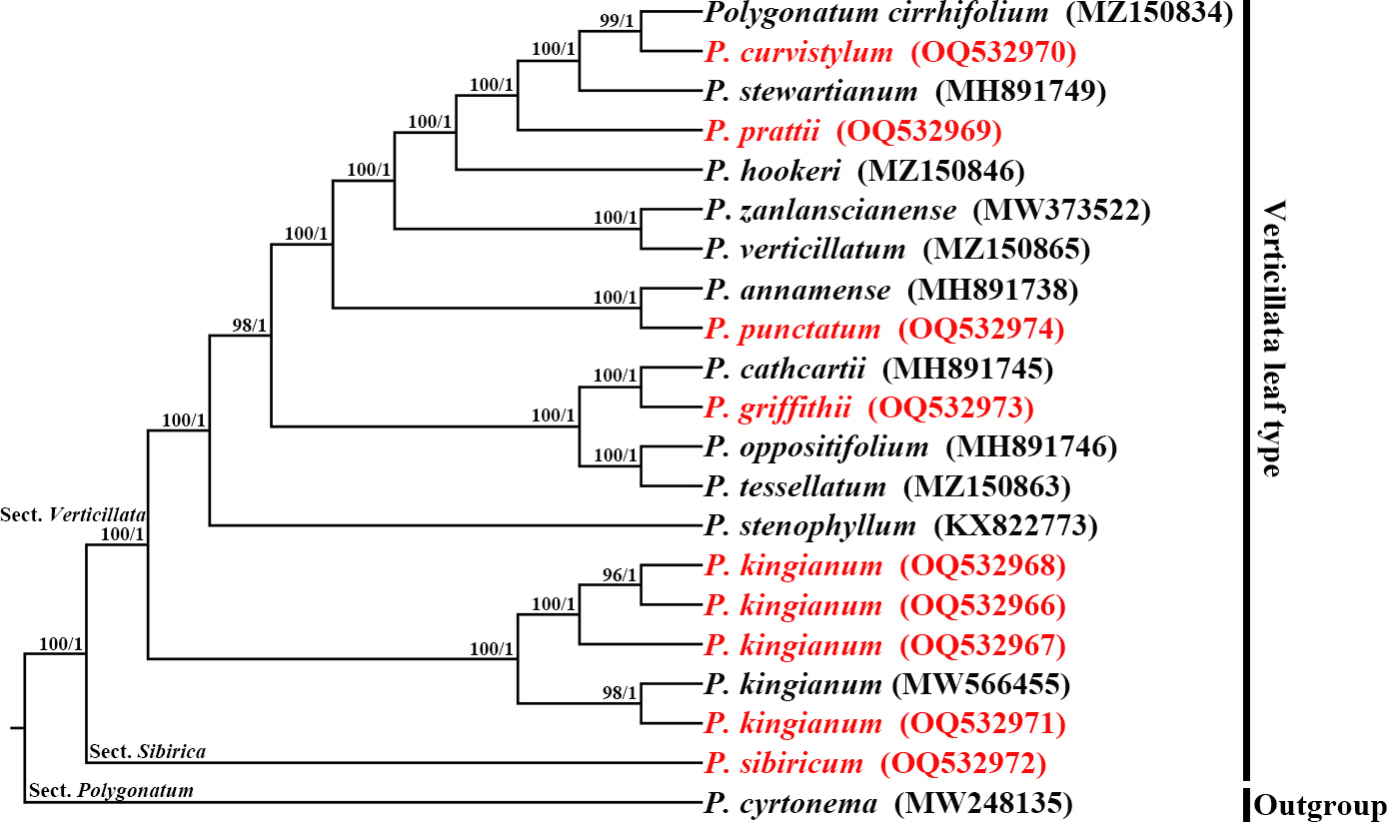
Phylogenetic reconstruction using Maximum likelihood (ML) and Bayesian inference (BI) methods, based on the chloroplast genomes sequences. The numbers above the branches indicate ML bootstrap values (BS)/BI posterior probabilities (PP).

We employed the whole cp genome sequences to build the phylogenetic tree to determine the phylogenetic relationship. Maximum likelihood (ML) and Bayesian inference (BI) were used to perform phylogenetic analyses with *P. cyrtonema* as an outgroup. The topologies of the ML and BI trees were identical (**Figure 7**). *P. kingianum* with different morphological characteristics clustered together with high support. *P. kingianum* was closely related to *P. stenophyllum* and *P. sibiricum* (BS = 100, PP = 1.00). *P. griffithii* and *P. cathcartii* clustered into a subclade and formed a sister relationship with the subclade of *P. oppositifolium* and *P. tessellatum*. *P. punctatum* and *P. annamense* clustered into a subclade; *P. verticillatum* and *P. zanlanscianense* clustered into a subclade; and *P. hookeri*, *P. prattii*, *P. stewartianum*, *P. curvistylum*, and *P. cirrhifolium* clustered into a subclade.

## Discussion

### Chloroplast genome structure

In this study, the cp genomes of nine samples were sequenced by next-generation sequencing (NGS) methods. Similar to previous studies (Huang X. et al., 2022; Zhang et al., 2022; Yang et al., 2023), the newly sequenced cp genomes of the samples were characterized, which displayed the typical quadripartite structure of flowering plants, and cp genomes contained more AT content than GC content. The nine cp genomes were similar both in size and composition. In the current study, we found that most of the *infA* genes in these cp genomes were pseudogenes except *P. sibiricum*. Pseudogenization is common in cp genomes ( Abdullah et al., 2021;Li X. et al., 2021; Scobeyeva et al., 2021). In the present study, base insertion was responsible for the pseudogenization of the *infA* genes. All in all, the cp genomes were highly conservative similar to the majority of plants.

### Analysis of highly variable regions

These cp genomes underwent a comparative analysis with sequences from Genebank. We compared the boundary between the SC and two IR regions and their neighboring genes. There was no significant difference at the boundary of most species except in the LSC and IR junctional regions of *P. sibiricum*. Previous research demonstrated that many *rps19* genes of the genus *Polygonum* were located entirely in the IR region (Wang et al., 2022; Xia et al., 2022). In contrast, the two *rps19* genes of *P. sibiricum* were located partly in the LSC Region, possibly due to IR contraction. Sequence divergence among plastid genomes comparisons based on mVISTA and nucleotide diversity showed that the low-identity region was mainly distributed in the non-coding region of the SC region compared with the IR region through multiple sequence alignment. Mutational hotspots of the cp genome are often developed to distinguish closely related species or genera, which can serve as specific DNA barcodes (Dong et al., 2012). Our study identified eleven regions with a higher evolution rate, namely, *ccsA*, *psbF*, *ycf1*, *psbM*, *rps19*, *rpl16*, *rrn4.5–rrn5*, *trnG-GCC–trnfM-CAU*, *ccsA–ndhD*, *atpA–atpF*, and *rps19–psbA*, all of which might be potentially used as candidate markers for identifying *P. kingianum* and its related verticillate leaf type of the *Polygonatum* species.

### Simple sequence repeat analyses

Cp microsatellites (cpSSRs) exist widely in cp genomes and have the advantages of codominant inheritance, high polymorphism, and abundance (Huang et al., 2018). Therefore, cpSSR markers were widely used for species authentication, evolutionary studies, population genetics, and so on (Dzialuk et al., 2009; Jayaswall et al., 2015; Singha et al., 2017; Mahajan et al., 2022).

In our study, we found that cpSSRs abundances in different species were varied. Even within the same species (*P. kingianum*), the number and type of cpSSRs were different. A or T mononucleotide repetition was the most common primary repetitive type. C or G mononucleotide repetition existed in some species and was rare in number. Previous studies have suggested that this might be due to the convenience of A-T conversion over G-C in the cp genome (Huang Y. et al., 2022). The number of cpSSRs of five kinds of SSRs were discovered: mononucleotide > dinucleotides > tetranucleotide > trinucleotide > pentanucleotide. The cpSSRs detected in this study would be helpful for the identification of the verticillate leaf type of the *Polygonatum* species, for germplasm resource evaluation, and for the conservation of *P. kingianum*.

### Amino acid abundance and codon usage

Codon usage biases are found in all eukaryotic and prokaryotic genomes and refer to the unequal use phenomenon of synonymous codons in an organism (Zhou et al., 2016). Codon usage biases are associated with the gene expression level and accuracy of the translation, gene length, gene translation initiation signals, protein amino acid composition, protein structure, tRNA abundance, mutation frequency and patterns, and GC composition (Romero et al., 2000; Xu et al., 2011). These results suggested that all individuals share similar codon usage patterns, with only slight differences in the number of codons and the RSCU value. The AT-rich bias was strongest in the third codon position, and the results of our study are similar to those of other species with cp genome codon usage biases (Huang et al., 2020; Dong et al., 2021; Tang et al., 2021). The research on codon preferences could help us provide a theoretical basis for codon modification of exogenous genes, accuracy of prediction about new members of the cp gene family, and identification of unknown genes.

### Species discrimination and phylogeny analysis

Phylogenetic analysis is of great significance for clarifying genetic relationships and species identification (Lan et al., 2022). Despite their morphological diversity, *P. kingianum* could be clustered into one clade by the phylogenetic tree based on cp genomes. We inferred that the limited geographical distribution, short-distance pollination by insects, and long-distance seed dispersal by birds might contribute to the frequent intraspecies gene flow of *P. kingianum*. These factors could decrease genetic differences among different morphological *P. kingianum*, improving the diversity and adaptability of the species. The cp genome of *P. griffithii* was reported for the first time in this study. The plastome phylogeny found that *P. griffithii* and *P. cathcartii* were closely related, which confirmed the speculation of FOC (Flora of China). All verticillate leaf types of the *Polygonatum* species in this research paper, including *P. kingianum*, have different degrees of overlap in geographical distribution, and many of them also have morphological intermediates types (e.g., *P. sibiricum*, *P. verticillatum*, *P. cirrhifolium*, and *P. zanlanscianense*). These species were morphologically indistinguishable. Our high-resolution phylogenetic trees not only distinguished these species but also provided some insight into the verticillate leaf type of the *Polygonatum* species relationship.

## Conclusions

In the past few years, phylogenetic data has accumulated rapidly and has been widely used to analyze phylogenetic relationships, species identification, and so on. Because of their great commercial value of”medicine food homology”, *P. kingianum* is deemed to be one of the most popular species in the *Polygonatum* genus. However, the molecular and phylogenetic aspects of *P. kingianum* and related verticillate leaf types of the *Polygonatum* species have not been well-researched. Hence, in this study, we compared and analyzed 20 cp genomes from 16 verticillate leaf types of the *Polygonatum* species, including 9 newly sequenced genomes from *P. kingianum, P. curvistylum, P. griffithii*, *P. prattii, P. punctatum*, and *P. sibiricum.* All the newly sequenced cp genomes have similar genome structures and features, which were shown to be conservative. We also identified 11 highly variable cp regions and 64–81 SSR molecular markers, which may serve as potential molecular markers for phylogenetic relationships as well as for the identification of the *Polygonatum* species. Phylogenetic analyses showed that cp genomes could provide sufficient information for distinguishing the medicinal plant *P. kingianum* from morphologically similar congeneric relatives, which also revealed accurate phylogenetic relationships. The findings have great potential for the enhancement of exploitation and conservation strategies of the germplasm resources of *P. kingianum*.

## Data availability statement

The datasets presented in this study can be found in online repositories. The names of the repository/repositories and accession number(s) can be found in the article/**Supplementary Material**.

## Author contributions

GW conceived the study; NS, KM, and PX collected and analyzed the data; NS, NZ, and LT wrote the manuscript. All authors contributed to the article and approved the submitted version.
